# Estimated postnatal *p,p’-*DDT and *p,p’-*DDE levels and body mass index at 42 months of age in a longitudinal study of Japanese children

**DOI:** 10.1186/s12940-020-00603-z

**Published:** 2020-05-11

**Authors:** Laurence Plouffe, Delphine Bosson-Rieutort, Lina Madaniyazi, Miyuki Iwai-Shimada, Kunihiko Nakai, Nozomi Tatsuta, Shoji F. Nakyama, Marc-André Verner

**Affiliations:** 1 Department of Occupational and Environmental Health, School of Public Health, Université de Montréal, Montreal, Canada; 2 Centre de recherche en santé publique, Université de Montréal, CIUSSS du Centre-Sud-de-l’Île-de-Montréal, Montreal, Quebec Canada; 3 Department of Management, Evaluation & Health Policy, School of Public Health, Université de Montréal, Montreal, Canada; 4 Institut national d’excellence en santé et en services sociaux (INESSS), Montreal, Québec Canada; 5 Centre for Health and Environmental Risk Research, National Institute for Environmental Studies, Tsukuba, Ibaraki 305-0053 Japan; 6 Department of Pediatric Infectious Disease, Institute of Tropical Medicine, Nagasaki University, Nagasaki, Japan; 7 Department of Development and Environmental Medicine, Tohoku University Graduate School of Medicine, Sendai, Miyagi Japan

**Keywords:** *p,p’-*dichlorodiphenyltrichloroethane (*p,p’-*DDT), *p,p’-*dichlorodiphenyldichloroethylene (*p,p’-*DDE), Body mass index (BMI), Postnatal exposure, Breast milk, Toxicokinetic modeling

## Abstract

**Background:**

Children are exposed to *p,p’-*dichlorodiphenyltrichloroethane (*p,p’-*DDT) and *p,p’-*dichlorodiphenyldichloroethylene (*p,p’-*DDE) through placental and lactational transfer. Some studies have suggested that early-life exposure to these compounds could lead to increased body mass index (BMI) during childhood. Our aim was to assess whether children’s exposure during the first 2 years of life is associated with BMI z-score in Japanese children at 42 months of age.

**Methods:**

We used data from a birth cohort (*n* = 290) of the Tohoku Study of Child Development. *p,p’-*DDT and *p,p’-*DDE levels were measured in breast milk samples collected 1 month after birth, and levels in children were estimated using a toxicokinetic model for three exposure periods (0–6 months, 6–12 months, 12–24 months). Associations between exposure estimates and BMI z-score at 42 months of age were assessed using multivariate linear regression models.

**Results:**

We found no significant association between levels of *p,p’-*DDT measured in breast milk or estimated in children and BMI z-score. However, we observed associations between estimated *p,p’-*DDE levels in girls during all postnatal exposure periods and BMI z-score; for each log increase in the estimated *p,p’-*DDE levels, BMI z-score increased by 0.23 (C.I. 95%: 0.01, 0.45) for the 0–6 months exposure period, 0.26 (C.I. 95%: 0.06, 0.47) for the 6–12 months exposure period, and 0.24 (C.I. 95%: 0.05, 0.43) for the 12–24 months exposure period.

**Conclusion:**

In this study of Japanese children, estimated postnatal *p,p’-*DDE levels were associated with increased BMI z-score at 42 months of age, mostly in girls. These results are in line with previous studies supporting that early-life exposure to *p,p’-*DDE may be associated with higher BMI during childhood.

## Background

*p,p’-*dichlorodiphenyltrichloroethane (*p,p’-*DDT) has mainly been used as an insecticide in agricultural crops. Although it was banned in most countries decades ago, it is still used in some countries for malaria vector control [1]. *p,p’-*DDT and its metabolites, such as *p,p’-*dichlorodiphenyldichloroethylene (*p,p’-*DDE), are widespread in the environment [2]. Both of these chemicals have been measured in human samples like breast milk [3–6].

Children are exposed to *p,p’-*DDT and *p,p’-*DDE through placental transfer and breastfeeding [7–9]. Because of their long biological half-lives and their lipophilic nature, *p,p’-*DDT and *p,p’-*DDE accumulate in the mother’s adipose tissues [10]. These chemicals can partition into breast milk lipids, making breastfeeding a major excretion route for the mother and a substantial exposure route for the child [11]. In most countries, adults’ exposure to *p,p’-*DDT and *p,p’-*DDE mainly comes from diet [12]. They are found in fatty foods such as red meat, poultry and eggs, dairy products and fish [11], the latter taking an important place in the Japanese diet. This could have an impact on Japanese children’s exposure, which occurs during a critical period of development.

Among all the health impacts of *p,p’-*DDT and *p,p’-*DDE, there has been an increasing interest in their relationship to obesity. A systematic review and meta-analysis conducted by Cano-Sancho et al. [13] gathered epidemiologic, in vivo and in vitro studies on the association between *p,p’-*DDT and *p,p’-*DDE and obesity. In vitro studies have shown that *p,p’-*DDT can impair adipose tissue homoeostasis and induce endocrine disruptions; results on *p,p’-*DDE were inconsistent. The in vivo studies found that perinatal exposure to *p,p’-*DDT can induce thermogenesis and lipid metabolism disruptions, especially in female offspring [14], and transgenerational obesity [15]. The meta-analysis of seven selected human studies revealed a significant positive association between *p,p’-*DDE exposure and higher BMI z-scores. Associations with other weight-related outcomes were less consistently reported, like associations between *p,p’-*DDT and BMI z-scores. The authors expressed the need for more epidemiologic and experimental studies, and classified these contaminants as “presumed” human obesogens.

Because most epidemiologic studies on the association of *p,p’-*DDT and *p,p’-*DDE with children’s weight focused on prenatal exposure, little is known about the potential impacts of exposure through breast milk. We only identified three studies on lactational exposure to *p,p’-*DDE and children’s weight [3, 16, 17]. Two out of three studies used approaches that do not account for the complexity of early-life pharmacokinetics, which are influenced by many factors such as child growth, breast milk content, and breastfeeding duration. Du et al. [3] used breast milk levels at 2, 5, 9 and 12 months in 16 mothers to estimate children’s exposure. They found no association between breast milk levels and infant growth and development measures at 12 months of age. Pan et al. [16] measured breast milk levels once at three months and multiplied this concentration with the breastfeeding duration to estimate children’s lactational exposure. They found no association between lactational exposure and infant growth at 12 months of age. As an alternative approach to assess lactational exposure, toxicokinetic modeling can be used to estimate children’s levels during and after lactation, overcoming the methodological limits of the Du et al. [3] and Pan et al. [16] studies. Such models have been developed [18–21] to estimate children’s levels during infancy/childhood, allowing the assessment of associations between exposure during hypothesized windows of vulnerability and various health outcomes. Iszatt et al. [17] used this approach to estimate prenatal and postnatal cumulative exposure to *p,p’-*DDE in seven European birth cohorts. They found an association between prenatal *p,p’-*DDE exposure and increased infant growth, but no association with postnatal exposure estimates. Given the limitations of previous studies and the paucity of data the association between lactational exposure to *p,p’-*DDT and *p,p’-*DDE and children’s weight, further investigation is warranted.

The aim of our study was to evaluate the association between measured breast milk levels and estimated children’s postnatal levels of *p,p’-*DDT and *p,p’-*DDE, and BMI z-score in 42-month-olds participating in the Tohoku Study of Child Development. Because no postnatal exposure assessment was conducted in this study, we used a validated toxicokinetic model to estimate children’s levels throughout their first two years of life.

## Materials and methods

### Study participants

The Tohoku Study of Child Development is a prospective birth cohort study that comprises two birth cohorts from an urban area and a coastal area cohort of northeastern Japan. The protocol and research areas have been described previously [22, 23]. The subjects of this study were the participants of the urban area study.

Pregnant women were recruited through their obstetrical units in two hospitals in the Sendai city [22]. Women were eligible if 1) they did not have any serious illness that could impair the fetus’ development; 2) they did not suffer from pre-eclampsia or gestational diabetes mellitus; 3) in vitro fertilization wasn’t used; 4) their native language was Japanese. Children’s inclusion criteria were 1) the absence of congenital anomalies or serious illnesses; 2) singleton birth, born after 36 weeks of gestation; 3) birth weight more than 2400 g, or higher than 2500 g if gestational age at birth was between 36 and 37 weeks. Written consent was obtained from all women.

Of the 1500 women who were met between January 2001 and September 2003, 687 gave their consent to participate in the study (participation rate: 46%). During their pregnancy, 88 women had to withdraw from the study, either because their baby did not meet the inclusion criteria (*n* = 54), because they moved to another region (*n* = 18), or because they dropped out or weren’t reachable (*n* = 16) [22, 23]. This narrowed the final number of participants to 599. We then selected the participants who had their breast milk tested for *p,p’-*DDT and *p,p’-*DDE, had data on total breastfeeding duration (highly influential parameter in the toxicokinetic model), and had data on child weight and height at 42 months (used to calculate BMI, the health outcome of this study), leaving 290 mother-child pairs.

All the procedures of this study were approved by the Medical Ethics Committee of the Tohoku University Graduate School of Medicine and of the National Institute for Environmental Studies [22]. In addition, the protocol for the analyses presented in this paper was approved by the Université de Montréal Institutional Review Board.

### Data collection

Data on demographics, lifestyle, medical history, and fish consumption was obtained through questionnaires administered four days after delivery [23]. Breastfeeding duration and anthropometric data such as children’s weight and height were assessed at the 7, 18, 30 and 42 months checkups through questionnaires. Only anthropometric measurements at 42 months were carried out on a large number of children by trained physicians or research coordinators using a certified electronic scale with 0.1 kg increments for weight and 0.1 cm increments for height (KS-110Mcp, Kansai-Seiki, Shiga, Japan). The measurement of body weight was performed without socks and heavy clothes.

### Chemical analysis

Mothers were asked to send a breast milk sample of more than 50 ml in a clean glass bottle one month after delivery. Samples were aliquoted in 10 ml centrifuge tubes and immediately frozen at − 80 °C until analyses.

All manipulations were made by IDEA Consultants, Inc. laboratory in Tokyo [23]. A sample of 5 ml of breast milk was mixed with 2 ml of saturated sodium oxalate, 20 ml of 1:1 v/v ethanol/hexane, and 10 ml of diethyl ether. The first hexane layer was mixed for 30 min, separated, and the residue was extracted twice with hexane. The hexane layer was then dehydrated using anhydrous sodium sulfate and evaporated. The lipid extract was weighted, dissolved in hexane and purified using Florisil cartridge column chromatography [24]. *p,p’-*DDT and *p,p’-*DDE were then measured using a gas chromatography/high-resolution mass spectrometry (HRGS/HRMS) system. The quality control was performed in accordance with the German external quality assessment scheme (G-EQUAS). The method limit of detection (30 pg/g-lipid for *p,p’-*DDT and 10 pg/g-lipid for *p,p’-*DDE) was calculated using the Currie et al. method [25]. Because the toxicokinetic model uses levels expressed on a lipid basis, we expressed concentrations in ng/g-lipid.

### Toxicokinetic model

**Fig. 1:**
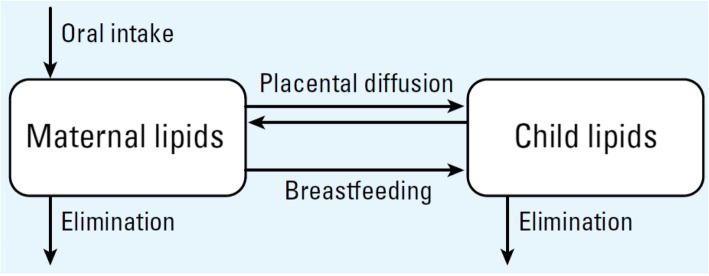
Conceptual representation of the toxicokinetic model. Reproduced from *Environmental Health perspectives* [18]

We used the toxicokinetic model of prenatal and lactational exposure to lipophilic persistent organic pollutants (including *p,p’-*DDT and *p,p’-*DDE) developed by Verner et al. [18] to estimate children’s levels. The model has two compartments: one for the mother’s lipids and one for the child’s lipids. The mother is exposed through diet and the child is exposed in utero through the placenta, and postnatally through breastfeeding (Fig. 1). The model assumes that lipophilic persistent organic pollutants are completely absorbed through the gastrointestinal tract and distribute evenly in lipids [18]. The percentage of lipids in fetal tissues was set to values at birth, and fetal and child growth was calculated using average growth curves [26]. The increase in maternal body fat mass during pregnancy was calculated as the difference between weight gain and increase in lean tissues. After delivery, the difference between pre-pregnancy weight and postpartum weight was attributed only to adipose tissue, which was assumed to be composed of 75% lipids [18]. Elimination rates were based on published half-lives (*p,p’-*DDT: ≈ 5 years [27] and *p,p’-*DDE: 13 years [28]). Exposure through breast milk in children was calculated based on average age-specific hourly milk intake and milk lipid content. The hourly breast milk intake for the first year of life was calculated using a formula based on data from Arcus-Arth et al. [29], and from Kent et al. [30] for the second year of life. We generated profiles of maternal, breast milk and children’s *p,p’-*DDT and *p,p’-*DDE levels for each participating mother-child pair. For each pair, the model incorporated individual-specific data on mother’s age at delivery, the total breastfeeding duration, mother’s body weight before pregnancy and one month after delivery, weight gain during pregnancy, and child’s sex. Available information did not allow estimating the duration of exclusive breastfeeding precisely. For that reason, only the total duration of breastfeeding was used, and daily breast milk intake was based on equations derived from studies including exclusively and partially breastfed children. For each pair and each chemical, iterative model simulations were performed to calibrate maternal lifetime daily oral intake until the breast milk level estimated with the model matched the breast milk level measured in the study. This maternal daily dose was then used in the model to estimate children’s areas under the concentration vs. time curve (AUCs) for three periods: 0 to 6 months, 6 to 12 months, and 12 to 24 months of age. Average children’s levels during these periods were calculated by dividing AUCs by the integration period. We performed toxicokinetic model simulations using acslX (Aegis Technologies Inc., Huntsville, AL, USA).

### Statistical analysis

Data was missing for 11% of the weight gain during pregnancy and 26% of the maternal weights one month after delivery. Missing data was due to non-response to the questionnaires. We tested the Missing at random assumption with t-tests for numeric variables, chi-squared test for categorical variables and an overall test of randomness (Little’s MCAR test). Most results for t-tests showed statistical significance (*p*-value < 0.05) but most of the chi-squared tests did not. Little’s MCAR test showed statistical significance. Thus, we concluded that the missing data was not missing completely at random (MCAR), but was missing at random (MAR) as there might be systematic differences between the missing and observed values, but these can be entirely explained by other observed variables [31]. Missing data related to the mothers’ body weight was imputed using the MICE multiple imputation package in R with predictive mean matching (pmm), and we set the number of imputed datasets to 100 [32, 33]. Toxicokinetic model simulations were performed for each mother-child pair, for each chemical, and for each of the imputed datasets.

The covariates were selected using a directed acyclic graph (DAG), an approach that reduces the degree of bias that can occur when measuring causal relationships between exposure and effect [34] (see Fig. S1 in Supplementary Material). Potential confounders included total breastfeeding duration (months, continuous [35];), weight gain during pregnancy (kg, continuous [36];), pre-pregnancy BMI (kg, continuous [36];), and fish intake (g/year, continuous [37];).

We calculated sex and age-specific BMI z-scores using the World Health Organization’s growth charts, which is an international standard taking into account children from different ethnic and cultural backgrounds [38]. Summary statistics were computed for our study outcome, exposures and covariates of interest. We performed correlation analyses to evaluate the relationship between measured (breast milk levels) and estimated (children’s levels) exposure metrics. Multiple linear regression analyses were performed to examine the relationship between exposure metrics (i.e., breast milk levels, estimated children’s levels for the 0–6, 6–12, and 12–24 months periods) and BMI z-scores at 42 months of age. Exposure metrics were ln-transformed prior to regression analyses. Two statistical models were evaluated. In Model 1, potential covariates identified in the DAG were kept in the regression models if they were correlated with the BMI z-scores and exposure metrics with a *p-*value < 0.2. In Model 2, all potential covariates identified in the DAG were included in the regression model. Analyses for both models were carried out including all children, and stratified by sex. We then looked at variance inflation factors (VIFs) to assess multicollinearity, and verified that the residual plots did not display unwanted patterns. Statistical analyses were performed in R, version 1.1.456 [32].

## Results

The median maternal age at delivery was 31 (range: 20–41) years, and the median total breastfeeding duration was 1.4 (range: 0.1–4.0) years (Table 1). Forty-four percent of the women obtained a college or vocational school degree, and 32% obtained a university degree. When missing, gestational weight gain and maternal body weight after pregnancy were imputed. The medians were similar in both the original and imputed datasets. Before pregnancy, 11% of the mothers were underweight, 85% were normal weight, 4% were overweight and 1% were obese according to the WHO BMI categories [39]. Excluded mother-child dyads tended to have a slightly higher maternal pre-pregnancy body weight and child birth weight. They also tended to smoke more than the included women. The large amount of missing data on ingested energy, on family income and on *p,p’-*DDT concentrations in breast milk in the excluded group made it impossible for us to assess the difference between included and excluded pairs for these variables.

**Table 1:** Characteristics of study participants

Variable	*n* (%)	Median (range)
Child characteristics		
Sex		
Male	156 (54)	
Female	134 (46)	
Gestational age (weeks)	290 (100)	40 (36, 42)
Birth weight (kg)	290 (100)	3.0 (2.2, 4.2)
42 month examination		
Weight (kg)	290 (100)	15 (11, 20)
Height (cm)	290 (100)	97 (89, 105)
BMI	290 (100)	16 (13, 20)
BMI z-score	290 (100)	0.5 (−2.0, 3.0)
Maternal characteristics		
Age at delivery (years)	290 (100)	31 (20, 41)
Height (cm)	290 (100)	158 (147, 173)
Weight (kg, pre-pregnancy)	290 (100)	52 (41, 81)
BMI (pre-pregnancy)	290 (100)	21 (16, 34)
Weight (kg, postpartum)	216 (75)	55 (42, 83)
Weight gain during pregnancy (kg)	258 (89)	9.3 (−1.4, 21.0)
Total breastfeeding duration (years)	290 (100)	1.4 (0.1, 4.0)
Education	290 (100)	
Junior high school	5 (2)	
High school	65 (22)	
College	127 (44)	
University	92 (32)	
Unknown	1 (0)	
Fish intake (g/year)	290 (100)	21,977 (0, 112,603)

Table 2 shows measured breast milk and estimated children’s levels. *p,p’-*DDT and *p,p’-*DDE were detected in all breast milk samples. The estimated children’s average concentrations for the three time windows (0–6 months, 6–12 months and 12–24 months) were estimated using the toxicokinetic model previously presented.

**Table 2:** Measured breast milk and estimated average children’s concentrations (ng/g-lipid) among participating children from birth to 24 months of age (*n* = 290)

Chemical	Min	P25	P50	P75	Max
*p,p’-*DDT					
Breast milk	1.4	4.4	6.3	8.6	34.5
Child 0–6 months	2.4	7.8	10.9	15.4	62.5
Child 6–12 months	3.0	10.7	14.6	21.7	98.9
Child 12–24 months	2.4	10.2	14.8	21.7	110.6
*p,p’-*DDE					
Breast milk	24.7	86.7	138.5	194.1	658.3
Child 0–6 months	44.4	156.1	247.4	357.5	1228.1
Child 6–12 months	46.4	216.5	349.3	513.3	1934.9
Child 12–24 months	48.0	222.9	370.3	551.6	2261.4

P: percentile.

Associations between *p,p’-*DDT and *p,p’-*DDE levels and BMI z-scores at 42 months are shown in Table 3. Model 1 represents the model adjusted only for maternal pre-pregnancy BMI, because it was the only covariate that respected our inclusion criteria. Model 2 is adjusted for all the variables we identified a priori as potential confounders using a DAG. We did not find any significant association with measured breast milk levels. Estimated children’s *p,p’-*DDT levels were not significantly associated with higher BMI z-scores in either model. Only one significant association was found with *p,p’-*DDE when including boys and girls; BMI z-score was positively associated with estimated children’s levels during the 12–24 months period. Additional statistically significant associations were revealed with sex stratification, with *p,p’-*DDE being associated with greater BMI z-score in girls for the three exposure periods in both Model 1 and Model 2. Overall, when significant associations were found, they were slightly stronger in Model 2. Beta coefficients for different time periods were similar, which may be related to the fact that exposure estimates were strongly correlated across periods, with correlation coefficients ranging from 0.87 to 0.98 for *p,p’-*DDT and 0.90 to 0.99 for *p,p’-*DDE. In all regression models, VIF values were below 1.2. Residuals were normally distributed, and showed no pattern indicative of non-linearity.

**Table 3:** Associations between ln-transformed measured breast milk or estimated children’s *p,p’-*DDT and *p,p’-*DDE levels and BMI z-score at 42 months of age. Statistically significant associations are presented in bold

	Model 1^a^				Model 2^b^			
	*p,p’-*DDT		*p,p’-*DDE		*p,p’-*DDT		*p,p’-*DDE	
	β (95% CI)	*p*	β (95% CI)	*p*	β (95% CI)	*p*	β (95% CI)	*p*
Measured breast milk concentrations								
All	−0.04 (− 0.23, 0.15)	0.706	0.08 (− 0.08, 0.24)	0.320	− 0.05 (− 0.24, 0.14)	0.629	0.07 (− 0.09, 0.24)	0.360
Girls^c^	0.11 (− 0.15, 0.36)	0.413	0.20 (− 0.03, 0.43)	0.085	0.10 (− 0.16, 0.35)	0.478	0.20 (− 0.02, 0.43)	0.078
Boys^c^	−0.18 (− 0.46, 0.09)	0.190	− 0.01 (− 0.24, 0.21)	0.901	−0.21 (− 0.49, 0.07)	0.141	−0.05 (− 0.28, 0.18)	0.680
Children’s area under the concentration vs. time curve (AUC) (0–6 months)								
All	−0.02 (− 0.21, 0.17)	0.847	0.09 (− 0.07, 0.25)	0.252	− 0.03 (− 0.21, 0.16)	0.790	0.09 (− 0.07, 0.25)	0.276
Girls^c^	0.15 (− 0.10, 0.40)	0.248	**0.23 (0.01, 0.45)**	**0.044**	0.13 (−0.12, 0.38)	0.313	**0.23 (0.01, 0.46)**	**0.042**
Boys^c^	−0.19 (− 0.47, 0.09)	0.174	− 0.02 (− 0.24, 0.20)	0.870	− 0.21 (− 0.49, 0.07)	0.138	− 0.05 (− 0.28, 0.18)	0.668
Children’s area under the concentration vs. time curve (AUC) (6–12 months)								
All	0.05 (− 0.12, 0.23)	0.547	0.14 (− 0.01, 0.29)	0.075	0.06 (− 0.11, 0.24)	0.480	0.15 (− 0.01, 0.30)	0.060
Girls^c^	0.21 (−0.02, 0.47)	0.069	**0.26 (0.06, 0.47)**	**0.011**	0.19 (−0.05, 0.43)	0.113	**0.28 (0.06, 0.49)**	**0.012**
Boys^c^	−0.12 (− 0.38, 0.14)	0.377	0.02 (− 0.21, 0.24)	0.871	− 0.10 (− 0.37, 0.17)	0.454	0.01 (− 0.21, 0.24)	0.904
Children’s area under the concentration vs. time curve (AUC) (12–24 months)								
All	0. 06 (− 0.10, 0.22)	0.474	0.14 (− 0.01, 0.28)	0.067	0.09 (− 0.08, 0.26)	0.309	**0.16 (0.01, 0.32)**	**0.034**
Girls^c^	0.19 (−0.02, 0.39)	0.070	**0.24 (0.05, 0.43)**	**0.012**	0.19 (−0.04, 0.42)	0.101	**0.27 (0.06, 0.48)**	**0.010**
Boys^c^	−0.09 (− 0.34, 0.15)	0.458	0.03 (− 0.19, 0.25)	0.810	− 0.05 (− 0.31, 0.21)	0.688	0.04 (− 0.18, 0.27)	0.702

^a^Adjusted for pre-pregnancy BMI

^b^Adjusted for pre-pregnancy BMI, weight gain during pregnancy, fish intake (g/year), and total breastfeeding duration

^c^Girls (*n* = 134); boys (*n* = 156)

## Discussion

In this study, we evaluated whether postnatal exposure to *p,p’-*DDT and *p,p’-*DDE is associated with BMI z-score in 42-month-old children participating in the Tohoku Study of Child Development in Japan. We found associations between children’s estimated *p,p’-*DDE levels and higher BMI z-scores (mostly in girls), but none with *p,p’-*DDT.

We selected BMI z-scores as our outcome because studies have shown that childhood obesity and/or overweight is associated with obesity, cardiometabolic morbidities and cancer in adulthood [40–42]. Associations were only observed with *p,p’-*DDE. This difference between chemicals could be due to a stronger obesogenic potential of *p,p’-*DDE than *p,p’-*DDT. However, this could also be due to the fact that measured *p,p’-*DDE levels of this contaminant were higher (possibility of greater analytical accuracy at higher levels), and that toxicokinetic model predictions were more precise and accurate for *p,p’-*DDE than *p,p’-*DDT in this cohort [18]. The mechanism of action of *p,p’-*DDT and *p,p’-*DDE on BMI increase remains uncertain. In vitro studies suggested that these chemicals may be endocrine disruptors [43], and that they could impair lipid secretion and metabolism [44]. In rat models, perinatal exposure to a commercial formulation of DDT can induce thermogenesis and lipid metabolism disruptions, especially in female offspring [14], and could induce transgenerational obesity [15]. The difference between sexes could be explained by the impact of *p,p’-*DDT and *p,p’-*DDE on the endocrine system, as the former is an estrogen and androgen receptor antagonist, whereas the latter is an androgen and progesterone antagonist [43]. Given the lack of data regarding sex-specific obesogenic mechanisms of *p,p’-*DDT and *p,p’-*DDE, the reasoning behind this phenomenon still remains uncertain.

In this study, we examined associations between estimated *p,p’-*DDT and *p,p’-*DDE levels during different time windows (0 to 6 months, 6 to 12 months, 12 to 24 months) and BMI z-score. Because the strength of the associations was similar from one time period to another, we weren’t able to identify a specific critical exposure window. This could be interpreted as the lack of a specific window of vulnerability to the obesogenic impacts of these chemicals during the first two years of life. Another explanation could be that estimated exposure metrics, being highly correlated across periods, did not allow distinguishing time-specific differences in strength of exposure-outcome associations.

Our effect estimates for postnatal exposure were slightly stronger than the meta-analytic effect estimate reported by Cano-Sancho et al. [13], but confidence intervals overlapped. All studies except one in the meta-analysis focused on prenatal exposure to *p,p’-*DDE. We found slightly stronger associations in Model 2 and with later exposure periods in general, although the differences were mostly negligible. In the meta-analysis, three studies stratified by sex to examine potential differences between boys and girls regarding associations with obesity indicators [45–47]. One of the studies focused on exposure at eight years of age and weight gain. They noticed an inverse association with BMI z-score in the highest exposed girls, and a positive association in the highest exposed boys, at 20 to 22 years of age [45]. Another study reported associations between prenatal exposure to *p,p’-*DDT and *p,p’-*DDE and higher BMI z-score at nine years of age in boys [46]. The third study found a significant association between prenatal exposure to *p,p’-*DDE and greater waist circumference and weight/height ratio in girls [47]. The meta-analysis concluded that no sex-specific trend could be identified with these results.

Our study has some important strengths. First of all, the longitudinal study design is ideal when evaluating associations between risk factors and developmental outcomes. Anthropometric data were collected directly by trained physicians or research coordinators using a certified electronic scale, which reduces measurement error. Typically, epidemiologic studies rely on one or few blood or breast milk levels to assess children’s early-life exposure to persistent organic pollutants like *p,p’-*DDT and *p,p’-*DDE. Given the complex changes in physiology and exposure during infancy, spot measurements are unlikely to provide comprehensive information on children’s exposure profile. Using our toxicokinetic model, we were able to reconstruct children’s levels from birth to two years, and to estimate cumulative exposure over three different periods (0–6 months, 6–12 months, 12–24 months). This allowed us to assess exposure-outcome associations during different potential windows of vulnerability.

Some limitations are also worth mentioning. First of all, when stratifying by sex, our sample size was cut by more than half, going from 290 to 134 mother-child pairs for analyses in girls. Also, estimations using the toxicokinetic model are not perfectly precise, and the error in level estimates likely impacted the precision in exposure-outcome models. Precise information on exclusive breastfeeding duration data wasn’t available, which could have potentially helped increase the precision of the estimates. Furthermore, uncertainty in measured breast milk *p,p’-*DDE and *p,p’-*DDT levels due to analytical measurement error and intra- and inter-feed variability may have impacted precision in exposure estimates. Calculating BMI z-scores based on WHO data may not be optimal for the Japanese population [48]; however, associations were similar when using BMI z-scores calculated using data from our study. We also didn’t take into account exposure to other environmental contaminants that could induce synergic, antagonistic or additive effects. We cannot exclude the possibility of residual confounding by unmeasured factors. Additionally, our study only included children who were breastfed for a month or more. Participants with missing information essential to our toxicokinetic model, such as breastfeeding duration and outcome measures in children, were excluded. The observed difference between included and excluded mother-child dyads, namely in terms of maternal pre-pregnancy body weight and smoking status, may decrease the generalizability of our results. Finally, correlations between the exposure metrics were very high, making it difficult to detect associations related to a specific exposure period.

## Conclusions

Our study suggested an association between estimated children’s *p,p’-*DDE levels and higher BMI z-scores in girls. These results suggest that prenatal exposure may not be the only sensitive time window when looking at chemical exposures and weight-related outcomes later in life. The strength of our associations concurred with that of Cano-Sancho et al.’s meta-analysis [13]. Postnatal exposure to chemicals should be taken into consideration when establishing strategies against the obesity epidemic and in the risk/benefit calculations of DDT usage in countries fighting malaria.

## Supplementary information

**Additional file 1: Fig. S1.** Directed acyclic graph (DAG) for the association between *p,p’-*DDT/E exposure metrics and BMI z-score at 42 months of age. **Table S1.** Correlations between breastfeeding duration and ln-transformed exposure metrics for *p,p’-*DDT. **Table S2.** Correlations between breastfeeding duration and ln-transformed exposure metrics for *p,p’-*DDE.

## Data Availability

To protect the confidentiality of study participants, data is not made publicly available. Please contact the corresponding author for data requests.

## Abbreviations

AUC: Area under the curve
BMI: Body mass index
DAG: Directed acyclic graph
*p,p’-*DDE: *p,p’-*dichlorodiphenyldichloroethylene
*p,p’-*DDT: *p,p’-*dichlorodiphenyltrichloroethane
