# Correction to: Estimated postnatal p,p’-DDT and p,p’-DDE levels and body mass index at 42 months of age in a longitudinal study of Japanese children

**DOI:** 10.1186/s12940-020-00609-7

**Published:** 2020-05-26

**Authors:** Laurence Plouffe, Delphine Bosson-Rieutort, Lina Madaniyazi, Miyuki Iwai-Shimada, Kunihiko Nakai, Nozomi Tatsuta, Shoji F. Nakayama, Marc-André Verner

**Affiliations:** 1 Department of Occupational and Environmental Health, School of Public Health, Université de Montréal, Montreal, Canada; 2 Centre de recherche en santé publique, Université de Montréal, CIUSSS du Centre-Sud-de-l’Île-de-Montréal, Montreal, Quebec Canada; 3 Department of Management, Evaluation & Health Policy, School of Public Health, Université de Montréal, Montreal, Canada; 4 Institut national d’excellence en santé et en services sociaux (INESSS), Montreal, Québec Canada; 5 Centre for Health and Environmental Risk Research, National Institute for Environmental Studies, Tsukuba, Ibaraki 305–0053 Japan; 6 Department of Pediatric Infectious Disease, Institute of Tropical Medicine, Nagasaki University, Nagasaki, Japan; 7 Department of Development and Environmental Medicine, Tohoku University Graduate School of Medicine, Sendai, Miyagi Japan

**Correction to: Environ Health (2020) 19:49**

**https://doi.org/10.1186/s12940-020-00603-z**

Following publication of the original article [1], the authors reported that one of the co-author’s last name was misspelled in the original article;

1.Incorrect name:

Shoji F Nakyama

2.Correct name:

Shoji F Nakayama

The original article has been corrected.
