# Supplementary material for: Estimated postnatal p,p’-DDT and p,p’-DDE levels and body mass index at 42 months of age in a longitudinal study of Japanese children
Source: Environ Health. 2020 May 11;19:49. doi: 10.1186/s12940-020-00603-z (PMC7216372; doi:10.1186/s12940-020-00603-z)
Supplement: Supplementary file 1 — Additional file 1: Fig. S1. Directed acyclic graph (DAG) for the association between p,p’-DDT/E exposure metrics and BMI z-score at 42 months of age. Table S1. Correlations between breastfeeding duration and ln-transformed exposure metrics for p,p’-DDT. Table S2. Correlations between breastfeeding duration and ln-transformed exposure metrics for p,p’-DDE. [file 12940_2020_603_MOESM1_ESM.docx]

## Supplementary material

**Title:** Estimated postnatal *p,p’-*DDT and *p,p’-*DDE levels in Japanese children and body mass index at 42 months of age

**Authors**: Laurence Plouffe, Delphine Rieutort, Lina Madaniyazi, Miyuki Iwai-Shimada, Kunihiko Nakai, Nozomi Tatsuta, Shoji F. Nakyama, Marc-André Verner

**Table of contents**

Figure S1. Directed acyclic graph (DAG) for the association between *p,p’-*DDT/E exposure metrics and BMI z-score at 42 months of age.

Table S1. Correlations between breastfeeding duration and ln-transformed exposure metrics for *p,p’-*DDT.

Table S2. Correlations between breastfeeding duration and ln-transformed exposure metrics for *p,p’-*DDE.

**Figure S1**. Directed acyclic graph (DAG) for the association between *p,p’-*DDT/E exposure metrics and BMI z-score at 42 months of age.


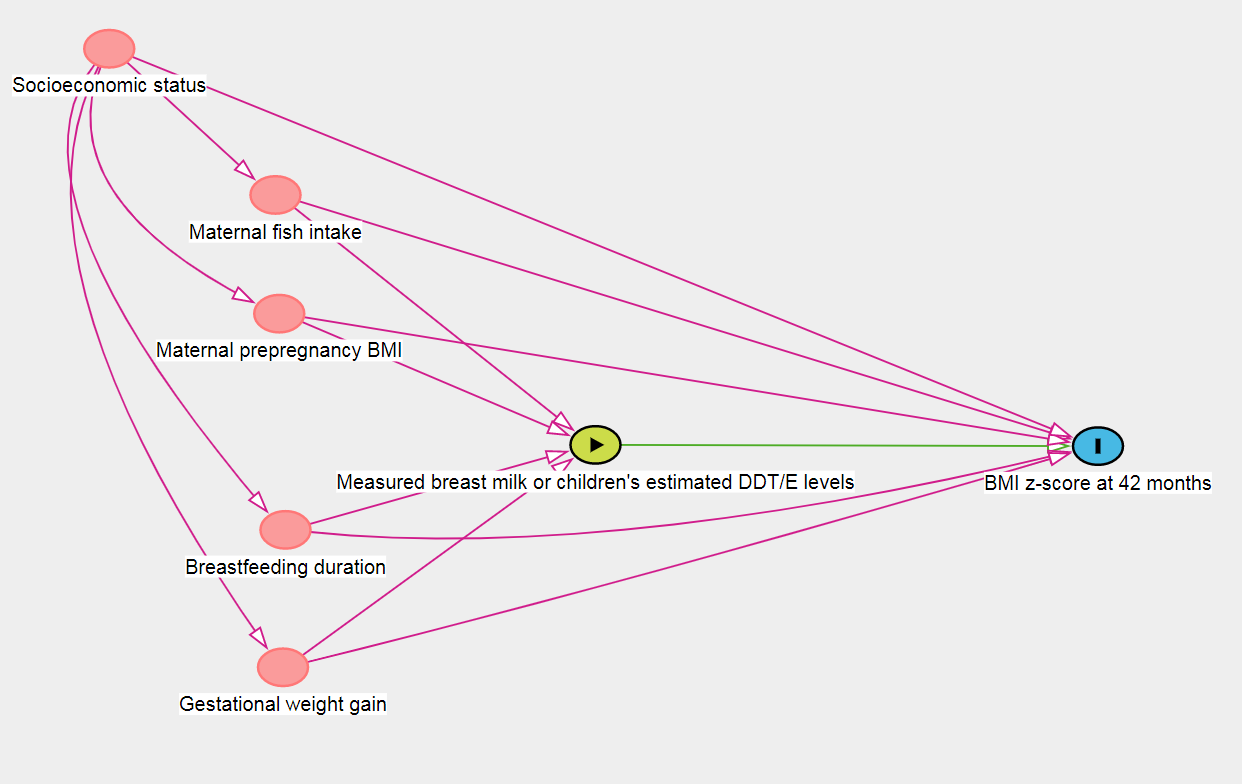


**Table S1.** Correlations between breastfeeding duration and ln-transformed exposure metrics for *p,p’-*DDT

| **Covariables** | **Breastfeeding duration** | **Breast milk concentration** | **AUC 0-6**  **months** | **AUC 6-12**  **months** | **AUC 12-24**  **months** | **Breast milk DDT x breastfeeding duration** |
| --- | --- | --- | --- | --- | --- | --- |
| **Breastfeeding duration** | 1 |  |  |  |  |  |
| **Breast milk concentration** | -0.079  (p=0.178) | 1 |  |  |  |  |
| **AUC 0-6 months** | -0.015  (p=0.794) | 0.992  (p<0.001) | 1 |  |  |  |
| **AUC 6-12 months** | 0.239  (p<0.001) | 0.899  (p<0.001) | 0.935  (p<0.001) | 1 |  |  |
| **AUC 12-24 months** | 0.395  (p<0.001) | 0.830  (p<0.001) | 0.872  (p<0.001) | 0.983  (p<0.001) | 1 |  |
| **Breast milk**  ***p,p’-*DDT x breastfeeding duration** | 0.721  (p<0.001) | 0.634  (p<0.001) | 0.678  (p<0.001) | 0.810  (p<0.001) | 0.883  (p<0.001) | 1 |

AUC: area under the curve

**Table S2.** Correlations between breastfeeding duration and ln-transformed exposure metrics for *p,p’-*DDE

| **Covariables** | **Breastfeeding duration** | **Breast milk concentration** | **AUC 0-6**  **months** | **AUC 6-12 months** | **AUC 12-24 months** | **Breast milk *p,p’-*DDE x breastfeeding duration** |
| --- | --- | --- | --- | --- | --- | --- |
| **Breastfeeding duration** | 1 |  |  |  |  |  |
| **Breast milk concentration** | -0.092  (p=0.116) | 1 |  |  |  |  |
| **AUC 0-6 months** | -0.039  (p=0.513) | 0.995  (p<0.001) | 1 |  |  |  |
| **AUC 6-12 months** | 0.182  (p=0.002) | 0.925  (p<0.001) | 0.951  (p<0.001) | 1 |  |  |
| **AUC 12-24 months** | 0.321  (p<0.001) | 0.871  (p<0.001) | 0.902  (p<0.001) | 0.987  (p<0.001) | 1 |  |
| **Breast milk *p,p’-*DDE x breastfeeding duration** | 0.650  (p<0.001) | 0.696  (p<0.001) | 0.731  (p<0.001) | 0.837  (p<0.001) | 0.896  (p<0.001) | 1 |

AUC: area under the curve
